# Rocking *Aspergillus*: morphology-controlled cultivation of *Aspergillus niger* in a wave-mixed bioreactor for the production of secondary metabolites

**DOI:** 10.1186/s12934-018-0975-y

**Published:** 2018-08-21

**Authors:** Tutku Kurt, Anna-Maria Marbà-Ardébol, Zeynep Turan, Peter Neubauer, Stefan Junne, Vera Meyer

**Affiliations:** 1 Department of Applied and Molecular Microbiology, Institute of Biotechnology, Technische Universität Berlin, Gustav-Meyer-Allee 25, 13355 Berlin, Germany; 2 Chair of Bioprocess Engineering, Institute of Biotechnology, Technische Universität Berlin, Gustav-Meyer-Allee 25, 13355 Berlin, Germany

**Keywords:** Single-use wave-mixed bioreactor, *Aspergillus niger*, Cyclodepsipeptide, Enniatin, Heterologous gene expression, Talcum microparticle, Tet-on system, Morphology, Macromorphology

## Abstract

**Background:**

Filamentous fungi including *Aspergillus niger* are cell factories for the production of organic acids, proteins and bioactive compounds. Traditionally, stirred-tank reactors (STRs) are used to cultivate them under highly reproducible conditions ensuring optimum oxygen uptake and high growth rates. However, agitation via mechanical stirring causes high shear forces, thus affecting fungal physiology and macromorphologies. Two-dimensional rocking-motion wave-mixed bioreactor cultivations could offer a viable alternative to fungal cultivations in STRs, as comparable gas mass transfer is generally achievable while deploying lower friction and shear forces. The aim of this study was thus to investigate for the first time the consequences of wave-mixed cultivations on the growth, macromorphology and product formation of *A. niger*.

**Results:**

We investigated the impact of hydrodynamic conditions on *A. niger* cultivated at a 5 L scale in a disposable two-dimensional rocking motion bioreactor (CELL-tainer^®^) and a BioFlo STR (New Brunswick^®^), respectively. Two different *A. niger* strains were analysed, which produce heterologously the commercial drug enniatin B. Both strains expressed the *esyn1* gene that encodes a non-ribosomal peptide synthetase ESYN under control of the inducible Tet-on system, but differed in their dependence on feeding with the precursors d-2-hydroxyvaleric acid and l-valine. Cultivations of *A. niger* in the CELL-tainer resulted in the formation of large pellets, which were heterogeneous in size (diameter 300–800 μm) and not observed during STR cultivations. When talcum microparticles were added, it was possible to obtain a reduced pellet size and to control pellet heterogeneity (diameter 50–150 μm). No foam formation was observed under wave-mixed cultivation conditions, which made the addition of antifoam agents needless. Overall, enniatin B titres of about 1.5–2.3 g L^−1^ were achieved in the CELL-tainer^®^ system, which is about 30–50% of the titres achieved under STR conditions.

**Conclusions:**

This is the first report studying the potential use of single-use wave-mixed reactor systems for the cultivation of *A. niger*. Although final enniatin yields are not competitive yet with titres achieved under STR conditions, wave-mixed cultivations open up new avenues for the cultivation of shear-sensitive mutant strains as well as high cell-density cultivations.

## Background

Filamentous fungi are of great economic importance as cell factories in industrial biotechnology. Due to their metabolic diversity, high production capacity, secretion efficiency, and the capability of conducting post-translational modifications, filamentous fungi like *Aspergillus niger* are widely exploited as cell factories for the production of organic acids, proteins and enzymes [1, 2]. Of interest is also their natural ability to synthesize bioactive secondary metabolites such as non-ribosomal peptides (NRPs) in large amounts [3, 4]. Due to the dramatic increase in the amount of pathogenic bacteria, which are resistant to commonly used antibiotics, the search for new antibiotics and other pharmaceuticals from fungal resources became one recent focus of the fungal research community [5–7].

An interesting class of bioactive fungal NRPs are the cyclodepsipeptides (CDPs) enniatin, beauvericin, bassianolide and PF1022, all of which consist of alternating units of *N*-methyl amino and α-hydroxy acids. CDPs exhibit antibacterial, antifungal, insecticidal, anthelmintic or even anticancer activities. Two of these compounds are commercialized drugs: fusafungine (a mixture of enniatins) is applied as antibacterial compound for treating throat infections in humans and emodepside (a semisynthetic derivative of PF1022A) used as anthelmintic compound in the veterinary medicine [8]. We showed recently that heterologous expression of the NRPS encoding gene *esyn1* from *Fusarium oxysporum* in *A.* *niger* results in enniatin B production in multigram scale per litre during a fed-batch cultivation [9]. A prerequisite to achieve such titres is controlled expression of the ESYN encoding gene *esyn1* under the Tet-on system [10] and feeding of the strain with the enniatin B precursors d-2-hydroxyvaleric acid (d-Hiv) and l-valine during cultivation. We also showed that this *esyn1* expressing strain DS3.1 became independent from d-Hiv feeding after introduction of the *kivR* gene from *F. oxysporum* into its genome (resulting in strain ÖV4.10). Constitutive expression of multiple copies of the *kivR* gene made ÖV4.10 competent to use its intracellular α-ketovaleric acid pool for d-Hiv generation. A direct comparison of both strains in 20 mL shake flask cultures revealed that the enniatin B titres achieved with strain ÖV4.10 are about 75% of the enniatin B titres of strain DS3.1 [9]. Polycistronic expression of both *esyn1* and *kivR* under control of the Tet-on expression system resulted in 40% of the product titres that were achieved with the strain DS3.1 in shake flask cultures. This finding proved that polycistronic secondary metabolite biosynthesis is possible in *A. niger* and suggested that the KivR enzyme catalyses the rate-limiting step in enniatin B biosynthesis [11]. It was furthermore demonstrated that *A. niger* is (i) a superior expression host not only for enniatin B, but also for beauvericin and bassianolide, by producing the highest titres ever reported for heterologous hosts as well as for natural producing organisms [12]; and (ii) an ideal platform strain for the production of new-to-nature CDPs obtained by designing and expressing hybrid CDP synthetases in *A. niger* [13, 14]. Indeed, some of the novel CDPs displayed considerably higher bioactivities compared to their parental CDPs and reference drugs [14]. The genetic (i.e. synthetic biology) tools to reprogram *A. niger* to overproduce bioactive non-ribosomal peptides at highest level have thus been successfully established and can be applied further to efficiently screen novel secondary metabolites from any fungal resource for desired bioactivities via heterologous expression of their encoding gene clusters in *A. niger*.

Apart from the necessity to discover and express novel bioactive compounds, another important technological development for their (industrial) production is the application of single-use bioreactors. These offer many opportunities for filamentous fungi as cell factories in pharmaceutical and cosmetics industries as recently highlighted in a White Paper of the EUROFUNG consortium [5]. Single-use bioreactors provide a platform technology, “which is safer (decreased risk of microbial contamination and cross-contamination), greener (reduced requirements for sterilization and cleaning), faster and more flexible (easy process and product changes), and cheaper (saving of time and costs)” [5]. In general, single-use bioreactors are reactor platforms that comprise of a non-disposable part of controllers, motor and housing, and a disposable bag, in which the cultivation is conducted. Several designs of single-use bioreactors were commercialized during the last decade. An increased process flexibility, high turnover and lower risk of cross contaminations led eventually to the penetration of this technology into the pharmaceutical industry [15, 16]. Several reactor types have been described and were compared with conventional non-disposable stirred tank bioreactors based on engineering parameters and process performances [17–22]. Single-use wave-mixed bioreactors are nowadays of interest for the cultivation of shear-sensitive cells, such as mammalian cell lines, insect cell lines [23], phototrophic algae [24] and plant hairy roots [25]. Also, successful cultivations with wave-induced agitation have been reported for the filamentous basidiomycetes *Flammmulina velutipes* and *Pleurotus sapidus* [26] as recently reviewed in [27].

So far, no reports on submerged cultivations of *A. niger* in single-use wave-mixed bioreactor systems have been published. *A. niger* and in general filamentous fungi adopt different microscopic and macroscopic morphological forms during submerged cultivations, the latter varying from a freely dispersed mycelium over loose mycelial clumps to dense pellets [28, 29]. Whereas dispersed mycelia increase medium viscosity and are sensitive to shear stress, oxygen and nutrient transfer is impaired in the core of pellets [30–32]. Process parameters, which affect the development of different macromorphologies range from inoculum concentration [33] and medium composition [31, 34] over reactor shape, stirrer geometry and stirrer speed (generating different shear forces) [35], aeration rate [36] to pH and temperature [37]. It was shown that macroscopic morphologies affect protein secretion rates in aspergilli in many, but not all cases [36, 38–47]. Unfortunately, the adequate macroscopic fungal morphology for given target products including organic acids, secreted proteins or secondary metabolites varies, cannot be generalized, and the advantages and disadvantages of mycelial or pellet cultivation have to be carefully adjusted for a given process and the final production scale [32, 35, 41, 48–57].

The main objective of this study was thus to determine whether a two-dimensional (horizontal and vertical) rocking-motion wave-mixed bioreactor is a viable alternative to well-established conventional STRs for the cultivation of *A. niger*. We therefore systematically analyzed for the first time the impact of controlled low shear stress cultivation on the growth of *A. niger*, the evolution of its macroscopic morphology and its product formation by cultivating the fungus in the wave-mixed bioreactor CELL-tainer. The two enniatin B-expressing strains DS3.1 and ÖV4.10 described above were used for this survey. They were cultivated under nutrient-limited fed-batch cultivation conditions with identical medium compositions, feeding schemes, temperature and pH control as described in our previously published STR cultivations [9] to allow a direct comparison. Naturally, we induced synthesis of enniatin B as model product via induction of the Tet-on expression system using the inductor doxycycline as previously described for STR cultivations [9].

## Results

**Fig. 1:**
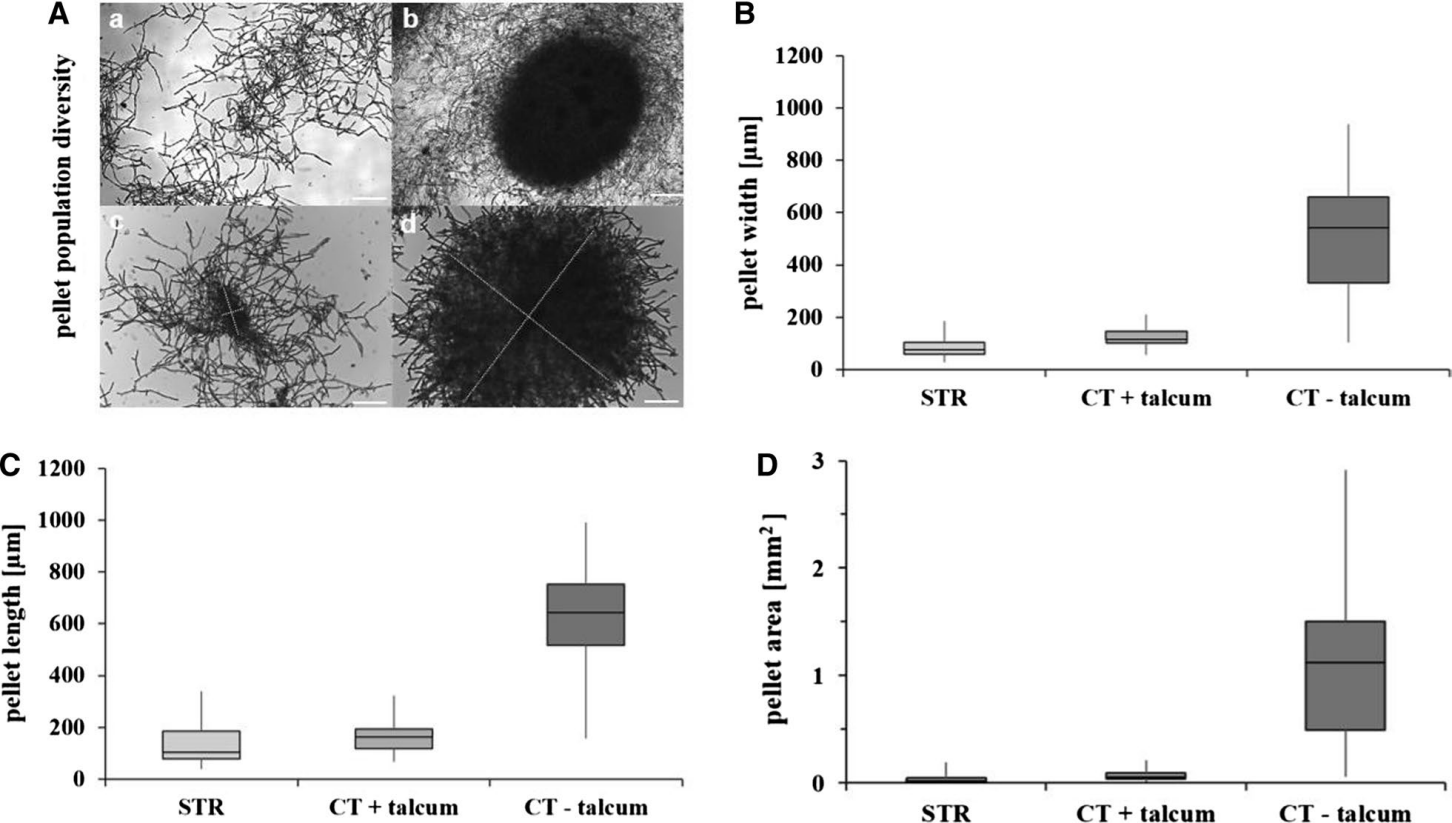
Macromorphological units observed during submerged fed-batch cultivations of *A. niger.* **A** Diversity of macromorphologies observed during STR (**a**) and CELL-tainer cultivations (**b**–**d**). Note that pellet formation could be prevented by addition of talcum microparticles to the medium (**c**). Only condensed regions of the pellets were considered for determination of their width and length (**c**, **d**). Scale bar: 100 µm. The pellet size was calculated by measuring the width (**B**) and the length (**C**) of more than 100 macromorphological units per cultivation run and calculating the area (**D**) with the formula *A *=* π width length*

The development of fungal macroscopic morphologies during submerged cultivations is a multifactorial process. Therefore, the final macromorphologies are unpredictable. However, from our experiences with STR cultivations of *A. niger* using BioFlo3000 reactors (5 L working volume, New Brunswick Scientific, NJ), we know that the chosen medium composition (see “Methods” section), a constant pH value of 3 and a stirrer speed of 750 rpm altogether ensure the formation of dispersed mycelia (Fig. 1A, panel a). Under these conditions, the formation of loose clumps or small pellets is a rare event (about 10–15%, Fig. 1A, panels b, c; [57, 58]). This is also observed for the enniatin B producing strain DS3.1 [9]. Note that shear forces in the CELL-tainer were lower at exactly the same operation conditions as applied in this study when compared to STR cultivations of the heterotrophic algae *Cryptecodinium cohnii*, which thus provoked less or even no cell lysis during operation [59, 60]. As the CELL-tainer system thus likely exhibits also less shear forces on *A. niger*, we first wished to analyse the macromorphologies from duplicate cultures of strain DS3.1, which followed exactly the same nutrient-limited fed-batch cultivation scheme as previously reported for the same strain in the BioFlo3000 reactor system [9]. Applying similar parameters under wave-mixed motion, however, lead to strong pellet formation of DS3.1 (Fig. 1A, panels b, d), which accounted for about 90% of macromorphological units (n > 100). The pellet population was highly diverse in shape (round–oval) and size (diameter between 300 and 800 μm). We thus measured both width and length of ~ 100 macromorphological units in STR and wave-mixed cultivations. Pellet sizes varied between 300 and 700 µm width, 500 and 800 µm length, which resulted in a projected area of 0.5–1.5 mm^2^ in wave-mixed cultivations (Fig. 1B–D). Note that some pellets displayed a highly condensed form, while some were surrounded by many loose filaments (Fig. 1A, panel b). Therefore, only the condensed part of each pellet was considered for the calculation of the pellet size.

Addition of talcum microparticles consisting of hydrous magnesium silicate to cultivation media was reported as a successful strategy to reduce pellet sizes of *A. niger* [32, 43]. The addition of 10 g L^−1^ talcum positively affected enniatin B production of strain DS3.1 in shake flask cultivations [9]. The addition of 10 g L^−1^ talcum to wave-mixed cultivations of the strains DS3.1 and ÖV4.10 decreased indeed pellet formation and led to macroscopic morphologies similar to STR cultivations (Fig. 1B–D and Table 1). The percentage of pellet-forming units decreased from 90% without talcum to 15% with talcum and the sizes of the macromorphological units were considerably reduced to values between 50 and 100 µm (width), 50 and 150 µm (length) and to values ≤ 0.1 mm^2^ (projected area).

**Table 1:** Growth and morphological parameters obtained from *A. niger* during submerged fed-batch cultivations

Strain *A. niger*	Reactor system	Cultivation mode	Pellet formation	Specific growth rate µ_max_ (h^−1^)	C_biomass,max_ (g_dryweight_ kg^−1^)	Reference
DS3.1	BioFlo3000	Fed-batch	+ (~ 15%)	0.15	24.9	[9]
DS3.1	CELL-tainer^®^	Fed-batch	+++ (~ 90%)	0.24	31.6	This study
DS3.1	CELL-tainer^®^	Fed-batch	+++ (~ 90%)	0.24	29.8	This study
DS3.1	CELL-tainer^®^	Fed-batch + talcum	+ (~ 15%)	0.11	34.0	This study
DS3.1	CELL-tainer^®^	Fed-batch + talcum	+ (~ 15%)	0.12	35.7	This study
ÖV4.10	CELL-tainer^®^	Fed-batch + talcum	+ (~ 15%)	0.11	27.7	This study
ÖV4.10	CELL-tainer^®^	Fed-batch + talcum	+ (~ 15%)	0.11	27.9	This study

**Fig. 2:**
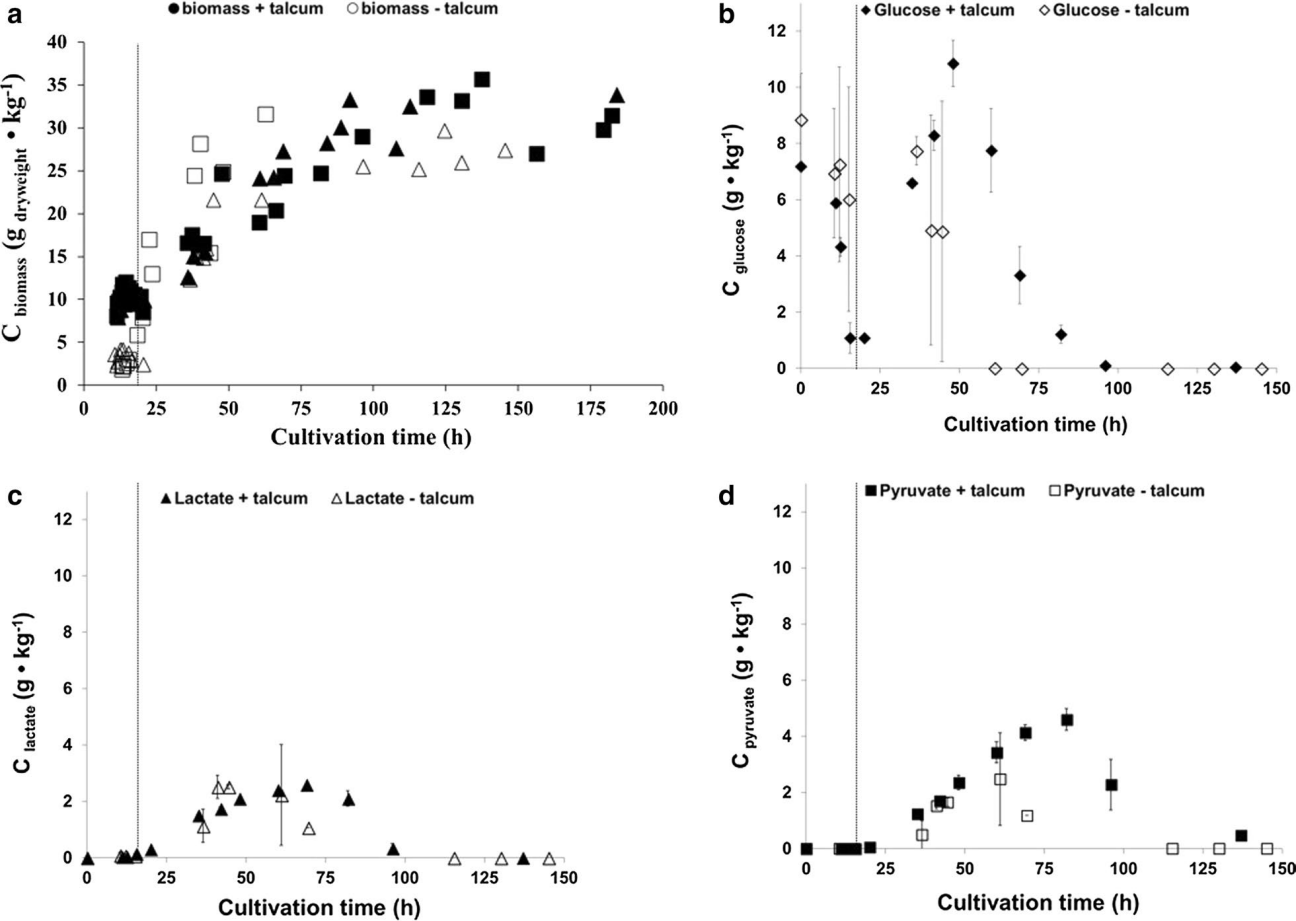
Growth and physiological profiles of duplicate *A. niger* cultures (strain DS3.1) during wave-mixed fed-batch cultivations. **a** Biomass titers produced by DS3.1 with talcum addition (closed symbols, replicates are represented by closed triangles and closed squares) and without talcum addition (open symbols, replicates are represented by open triangles and open squares). **b** Glucose concentrations in cultivations with (closed symbols) and without (open symbols) talcum. **c** Lactate concentrations in cultivations with (closed symbols) and without (open symbols) talcum. **d** Pyruvate concentrations in cultivations with (closed symbols) and without (open symbols) talcum. Start of glucose-feed and time of induction of Tet-on driven *esyn* expression via doxyxycline addition is indicated with a dashed line

**Fig. 3:**
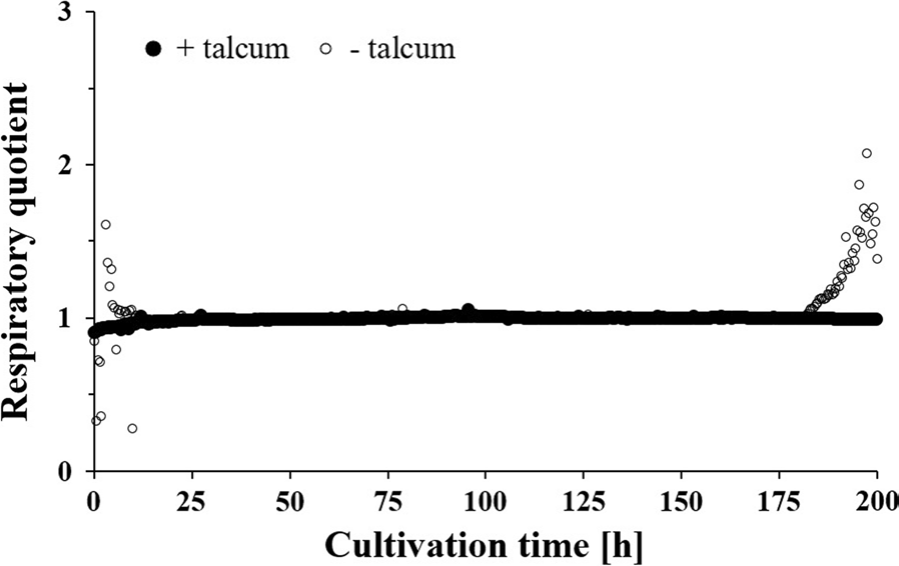
Evolution of the respiratory quotient during wave-mixed fed-batch cultivations of *A. niger* strain DS3.1. Cultivations supplemented with talcum (closed symbols) achieved a constant RQ of 1 throughout cultivation. In absence of talcum (open symbols), the RQ value varied between 0.3 and 1.6 within the first 10 h of cultivation and during the last 20 h of cultivation

Notably, the addition of talcum to DS3.1 and ÖV4.10 wave-mixed cultivations led also to reproducible growth curves, which was not the case for wave-mixed cultivations of DS3.1 without the addition of talcum (Fig. 2a). In the latter, high variations in physiological parameters (glucose and oxygen consumption, carbon dioxide production, biomass accumulation) and (by-)product formation (enniatin B, pyruvate, lactate) were observed (Figs. 2b, d, 3). This was likely a consequence of the high morphological variability of DS3.1 growth units. The respiratory quotient RQ, which is the ratio of the volumetric carbon dioxide production rate ${\text{Q}}_{{{\text{CO}}_{ 2} }}$ to the volumetric oxygen uptake rate ${\text{Q}}_{{{\text{O}}_{ 2} }}$, differed considerably between the wave-mixed cultivations if talcum was added or not. The RQ value is a good indirect measure for the physiological state of a culture and independent of its cell number. During growth on carbohydrates, an RQ value of 1 is an indicator for balanced aerobic metabolism, whereas an RQ value differing from 1 suggests metabolic discrepancies or even metabolic stress. DS3.1 cultivations, if supplemented with talcum, achieved a constant RQ of 1 throughout the whole cultivation time (200 h, Fig. 3). If cultivated in absence of talcum, however, the RQ value varied between 0.3 and 1.6 within the first 10 h of cultivation before it reached a constant value of 1 until 180 h. During the last 20 h, it increased to a value of 2 due to a slightly faster decreasing ${\text{Q}}_{{{\text{O}}_{ 2} }}$ (Fig. 3). Most interestingly, DS3.1 cultivated under wave-mixed conditions reached higher maximal specific growth rates (0.24 h^−1^) in the absence of talcum when compared to its cultivation in the presence of talcum (0.11 h^−1^), but lower final biomass titres (~ 30 g kg^−1^ compared to ~ 35 g kg^−1^; Fig. 2a and Table 1). Faster growth of DS3.1 in the absence of talcum became also evident when the concentration of glucose and the by-product lactate were measured in culture samples at different time points. As depicted in Fig. 2c and d, glucose and lactate depleted already after ~ 60 h in the DS3.1 cultivations without talcum addition, but only after ~ 90 h in the cultivations with 10 g L^−1^ talcum. Since the same amount of nutrients were available in both cases, a lower concentration of biomass and lactate has to result in the accumulation of other by-products, which are not re-assimilated for growth or main product formation at the later stage of the cultivation. Altogether, these observations suggest that morphology engineering via talcum addition decreases and homogenises pellet sizes. This however, leads to a lower specific growth rate and slower byproduct accumulation.

**Fig. 4:**
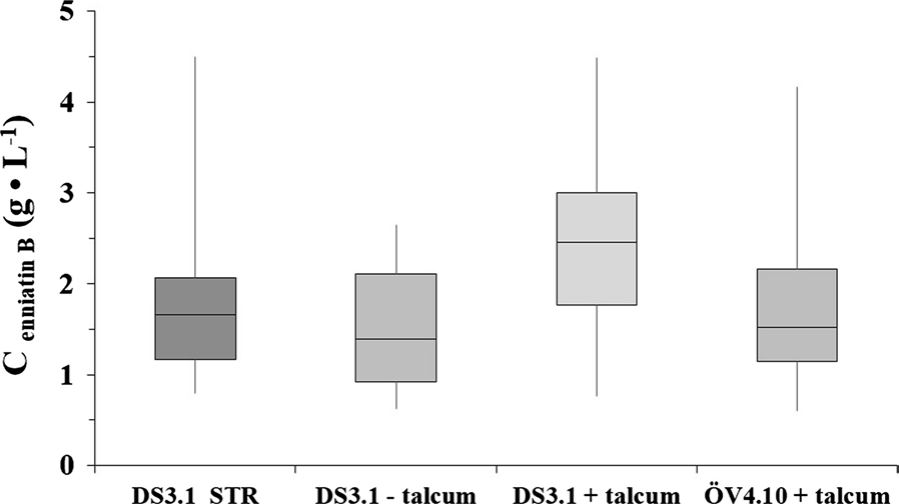
Enniatin B titres during wave-mixed fed-batch cultivations obtained from the *A. niger* strains DS3.1 and ÖV4.10. Volumetric enniatin B yields (g L^−1^ _culture broth_) from duplicate cultures are given, which were measured as technical replicates. Enniatin B was determined after 100 h of each cultivation approximately every 10 h. Biological and technical replicates are summarized in each box–whisker-plot

Enniatin B titres measured in DS3.1 and ÖV4.10 biomass samples harvested in the CELL-tainer system in the presence or absence of talcum after 100 h of fed-batch cultivation are depicted in Fig. 4. We decided to display the data in a box–whisker plot graphic as enniatin B values usually sway a lot in biological and technical duplicates. As expected, DS3.1 surpasses strain ÖV4.10 regarding enniatin B production in compliance with our previous findings in shake flask cultivations [9]. The data also demonstrate that volumetric as well as specific enniatin B yields are higher in DS3.1 cultures when cultivated in the presence of talcum (2.5 g kg^−1^/0.08 g g^−1^ DW) compared to cultivations in the absence of talcum (1.4 g kg^−1^/0.055 g g^−1^ DW), indicating that smaller pellet sizes and slower growth are advantageous for enniatin B production. However, the enniatin B yields of DS3.1 in wave-mixed cultivations are lower compared to the yield obtained in the STR (0.18 g g^−1^ DW) [9].

## Discussion

Increased process flexibility, high turnover and lower risk of cross-contaminations in single-use bioreactor systems underpin the importance of this technology for the pharmaceutical industry [15, 16]. Another advantage is the design flexibility that made different geometries available. The aim of this study was to investigate the impact of low shear forces generated in the single-use wave-mixed CELL-tainer on growth, development of macroscopic morphologies and enniatin B product formation in *A. niger* and to compare the data with physiological and morphological data obtained for *A. niger* STR cultivations in a BioFlo3000 STR.

A critical parameter for any process design for aerobic cultivations is the volumetric oxygen transfer coefficient k_L_a of the reactor. The k_L_a value describes the capacity of a given system to introduce oxygen into the liquid phase. To increase the k_L_a, vigorous stirring or shaking is necessary to break up air bubbles, thereby increasing the area of the liquid–gas interface. Stirring is the main reason for the high shear stress in STRs. While one-dimensional rocking reactors exhibit relatively low k_L_a values of 1 to 5 h^−1^, k_L_a-values beyond 400 h^−1^ were reported for wave-mixed bioreactors [17, 27], similar to values obtained in lab-scale stirred tank reactors with stirring rates as applied in this study. The CELL-tainer concept was thus successfully applied for aerobic fed-batch cultivations of *Escherichia* *coli,* in which typical growth rates and recombinant protein expression levels were achieved [17]. Notably, specific growth rates for *A. niger* cultivated under identical nutrient-limited fed-batch cultivations under wave-mixed conditions are considerably higher (0.24 h^−1^) in comparison to STR cultivations (0.15 h^−1^), indicating that high shear forces under stirred agitations indeed limit the growth of *A. niger*. It is conceivable that reduced shear stress under wave-mixed conditions allows self-assembly of *A. niger* growth units, which generate the natural architecture for a very heterogeneous pellet population, which is eventually important to ensure fast nutrient uptake, and thus biomass accumulation. This data also suggests that macromorphological pellet heterogeneity of *A. niger* can be beneficial to the growing population to grow as fast as possible. This hypothesis is highly speculative and has not been studied yet in filamentous fungi; however, many studies, which investigated bacterial population heterogeneities indeed propose that even single cells follow a population-based strategy to allocate different tasks to different cells (e.g. to specialise on different metabolic pathways) in order to maximise population growth [61–63]. Given that macromorphological pellet heterogeneity provides a fitness advantage to *A. niger*, one would expect that this advantage will get lost when the pellet size becomes homogeneous. This is exactly what we have observed after the addition of talcum to wave-mixed cultures: the specific growth rate dropped to 0.11 h^−1^ and glucose was metabolised much slower (Figs. 1, 2 and Table 1). On the one hand, talcum microparticles could probably interfere with important physical factors that control the self-organisation of growth units, especially in early growth phases (note that RQ variations during the first 10 h were only evident for wave-mixed cultivations lacking talcum; Fig. 3), on the other hand, talcum microparticles might cause additional friction stress to *A. niger*. We are currently studying this hypothesis further on the molecular level.

In conclusion, CELL-tainer cultivations seem to be superior for fast biomass accumulation of *A. niger* and could thus be interesting for growth-coupled product formation such as primary metabolites (organic acids) or even secreted proteins. The synthesis of enniatin B, however, is linked to secondary metabolism of *A. niger*. We showed previously that reduced growth rates and even carbon starvation are beneficial for higher enniatin B yields, likely because it generally activates secondary metabolism of *A. niger* [9, 58, 64]. Hence, one would expect lower enniatin B yield in fast-growing cultures like in wave-mixed cultivations of DS3.1 lacking talcum compared with slow-growing wave-mixed cultures of DS3.1 with talcum. This is indeed what we have observed in this study (Fig. 4).

## Conclusions

In this study, we present a single-use wave-mixed bioreactor concept as an alternative to well-established conventional STR cultivations for *A. niger* for the first time. The CELL-tainer system can ensure higher specific growth rates than in the STR cultivations and, interestingly, does not require the addition of antifoam agents as no foam formation was observed (data not shown). It thus makes the addition of antifoaming agents needless, as previously reported for wave-mixed bioreactor systems [18]. We thus conclude that this reactor system has a very high potential for growth-coupled production processes for *A. niger*, in which the formation of macromorphologies should remain unrestricted. For secondary metabolite production, however, wave-mixed cultivation needs to be further optimized with respect to controllable pellet morphologies.

## Methods

### Strains and media

*Aspergillus* strains used in this study are summarized in Table 2 and were previously described [9]. Spores of *A. niger* strains were obtained and harvested as described in Refs. [9, 11] and used freshly to inoculate bioreactors.

**Table 2:** *A. niger* strains used in this study

Strain	Relevant genotype	References
DS3.1	Tet-On-*esyn1*-T_*trpC*_, *pyrG*^+^, single copy	[9]
ÖV4.10	Tet-On-*esyn1*-T_*trpC*_, *pyrG*^+^, single copy; P_gpdA_-e*kivR*-T_*trpC*_-*amd*S	[9]

### Bioreactor cultivation

Submerged cultivations were performed with 6.6 L BioFlo3000 bioreactors (New Brunswick Scientific, NJ, USA) and in the 20 L disposable wave-mixed bioreactor CELL-tainer^®^. The main fermentation settings were already described in Refs. [9, 58, 64]. In brief, fed-batch cultivations were inoculated with spore suspension of *A. niger* strains with a spore titre of 10^9^ conidia L^−1^ and 0.003% yeast extract to improve germination. Initial batch cultivations were conducted with 4 L of cultivation medium, containing 0.8% glucose (w v^−1^). When reaching the late exponential growth phase, the Tet-on system was induced by addition of 20 µg mL^−1^ doxycycline (Dox). At the same time, the feed was started with a feeding rate of F = 0.046 L h^−1^. The feed medium (1.5 L) contained 5% glucose, 0.5% yeast extract, 0.1% casamino acids, 20 mM d-2-hydoxyvaleric acid (d-Hiv) and 20 mM l-valine for DS3.1 and 40 mM l-valine for ÖV4.10 cultivations additionally to the initial fermentation medium. In total, 100 µg mL^−1^ of Dox were added to the culture (20 µg mL^−1^ of Dox addition every 4–7 h). A temperature of 30 °C was kept constant throughout the whole cultivation process and a pH-value of 3.0 was maintained by addition of 2 M NaOH or 1 M HCl, respectively. The aeration rate was kept constant at 1 L min^−1^.

The rocking motion of the CELL-tainer was initially set to 5 rpm during the germination phase of 5–6 h. The rocking speed was controlled between 15 and 45 rpm after germination, depending on the dissolved oxygen (DO) value output (DO-controlled rpm mode).

### Metabolite and gas analysis

Metabolite analysis of the supernatant samples (i.e. carbohydrates and short chain carboxylic acids) was conducted with a refractometric detector on HPLC systems as previously described [65]. Enniatin extraction and measurements were performed as previously described [9]. In brief, defined amounts of biomass were subjected to ethyl acetate extraction, centrifuged and evaporated. Samples were dissolved in 40% isopropanol, diluted 10- to 10,000-fold and enniatin concentration quantified by ESI-Triple-Quadrupol-MS (6460 Series, Agilent Technologies) analysis in the multiple reaction monitoring mode [9]. Enniatin isolated from *F. oxysporum* was used as an external standard to generate a calibration curve out of the manually integrated peak areas for each measurement. The O_2_ and CO_2_ content in the off-gas was measured with the Blue-In-One analyser (Bluesens).

### Microscopy and determination of pellet size

Mycelia and pellets were stained with lactophenol blue and analysed with differential interference contrast (DIC) microscopy using Leica DM 5000 CS and evaluated using Leica’s microscope software LAS V4.3. Image analysis of the pellet width and length was performed using ImageJ 1.51p. Disperse mycelia, loose clumps or dense pellets were each defined as macromorphological unit given that they were individually distinguishable by microscopy from each other. More than hundred macromorphological units were analysed per sample.

### Equations

The specific growth rate µ was determined by plotting the logarithm of the biomass against the cultivation time during exponential growth. Respiratory quotient (RQ), volumetric oxygen uptake rate (${\text{Q}}_{{{\text{O}}_{ 2} }}$) and volumetric carbon dioxide production rate (${\text{Q}}_{{{\text{CO}}_{ 2} }}$) were calculated following Eqs. (1), (2), (3).

1 $$Q_{{O_{2} }} = \frac{{\dot{V}_{G}^{\alpha } }}{{V_{F} \cdot 22.4}}\left[ {Y_{{_{{O_{2} }} }}^{\alpha } - \frac{{1 - Y_{{_{{O_{2} }} }}^{\alpha } - Y_{{_{{O_{2} }} }}^{\alpha } }}{{1 - Y_{{_{{O_{2} }} }}^{\omega } - Y_{{_{{CO_{2} }} }}^{\omega } }} \cdot Y_{{_{{O_{2} }} }}^{\omega } } \right]$$

2 $$Q_{{CO_{2} }} = \frac{{\dot{V}_{G}^{\alpha } }}{{V_{F} \cdot 22.4}}\left[ {Y_{{_{{CO_{2} }} }}^{\omega } \cdot \frac{{1 - Y_{{_{{O_{2} }} }}^{\alpha } - Y_{{_{{O_{2} }} }}^{\alpha } }}{{1 - Y_{{_{{O_{2} }} }}^{\omega } - Y_{{_{{CO_{2} }} }}^{\omega } }} - Y_{{_{{CO_{2} }} }}^{\alpha } } \right]$$

3 $$RQ = \frac{{Q_{{CO_{2} }} }}{{Q_{{O_{2} }} }}$$

### Data fitting

Off-gas data were fitted with the fitting toolbox of SigmaPlot TableCurve 2D v5.01 (Systat Software Inc.).
